# A Facile One-pot Synthesis of 1-Arylpyrazolo[3,4-d]Pyrimidin- 4-ones

**DOI:** 10.3390/molecules15053079

**Published:** 2010-04-27

**Authors:** Xiaohong Zhang, Qiulian Lin, Ping Zhong

**Affiliations:** 1 Oujiang College, Wenzhou Universtity, Wenzhou 325035, China; 2 College of Chemistry and Materials Engineering, Wenzhou Universtity, Wenzhou 325035, China; E-Mails: kamenzxh@163.com (X.H.Z.); xiaoxiaolinrong@163.com (Q.L.L.)

**Keywords:** POCl_3_, one-pot, RCOOH, pyrazolo[3,4-d]pyrimidine

## Abstract

One pot synthesis of 1-arylpyrazolo[3,4-d]pyrimidin-4-ones by the reaction of 5-amino-N-substituted-1*H*-pyrazole-4-carbonitrile with different lower aliphatic acids in the presence of POCl_3_ has been developed_._ The structures of all the title compounds have been confirmed by IR, ^1^H-NMR,^ 13C^-NMR, and elemental analyses. Moreover, the structures of one of these compounds, **2c**, was confirmed by single-crystal X-ray diffraction.

## 1. Introduction

**Scheme 1:**
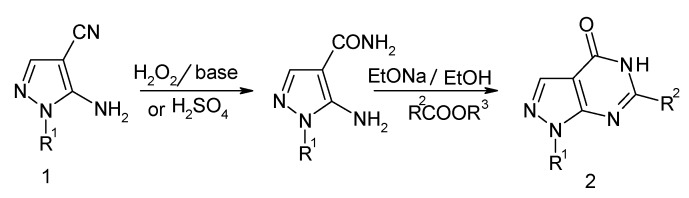
Synthesis of pyrazolo [3, 4-*d*] pyrimidin-4-ones by the reaction of esters.

**Scheme 2:**
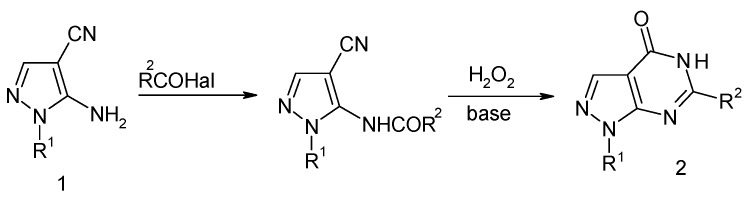
Synthesis of pyrazolo [3, 4-*d*] pyrimidin-4-ones by the reaction of acyl chlorides.

Pyrazolopyrimidinone derivatives have attracted the attention of numerous researchers over many years due to their important biological activities [1,2,3,4]. Structural analogs of pyrazolo[3,4-*d*]- pyrimidines have displayed good activities as inhibitors of cyclin-dependent kinase 2 [5] and PI3 kinase-A [6], anticancer and radioprotective activity [7], antimicrobial [8] and other biology activity [9]. The importance of pyrazolo[3, 4-*d*]pyrimidines had resulted in the development of several synthetic methods for their construction [10,11]. The traditional transformation utilizes two steps to assemble aminopyrazolo[3, 4-*d*] pyrimidin-4-ones, as illustrated in Scheme 1 and Scheme 2. However, the transformation of compounds **2** requires two steps and sufferes from several disadvantages such as vigorous conditions, long reaction times and low yields [12,13]. The development of one-step and efficient syntheses of aminopyrazolo[3,4-*d*]pyrimidin-4-ones under mild conditions remained a work in progress.

Here, we report a simple and efficient method for the synthesis of usefully functionalized pyrazolo[3,4-d] pyrimidins-4-ones **2** by heteroannulation under mild conditions using POCl_3_.

## 2. Result and Discussion

**Scheme 3:**
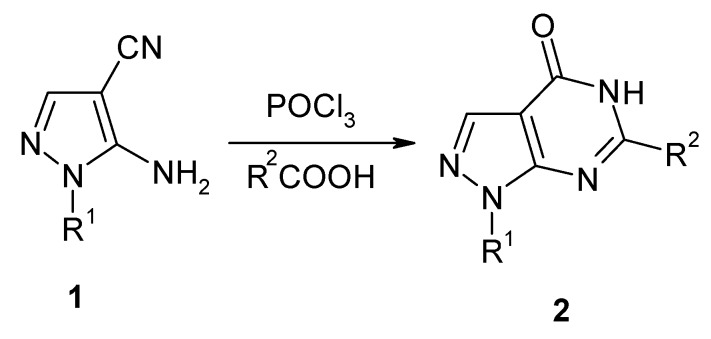
Synthesis of pyrazolo[3, 4-*d*]pyrimidin-4-ones by the reaction of carboxylic acid in the presence of POCl_3_.

The 5-amino-N-substituted-1H-pyrazole-4-carbonitrile starting materials **1**, synthesized by a one–pot synthesis literature procedure [14], was then reacted with lower aliphatic acids in the presence of POCl_3_ to give the target N-substituted pyrazolo[3,4-*d*]pyrimidin-4-ones **2** (Scheme 3). 

A number of works about POCl_3_-catalyzed reactions, especially intramolecular condensations [15] have been reported. In our reaction system POCl_3_ acted not only as a chlorinating reagent, but also an oxidant. Thus, we concluded that the 5-amino-N-substituted-1*H*-pyrazole-4-carbonitrile were first oxidized to give the corresponding N-substituted-5-amino-pyrazole-4-carboxamide, which immediately reacted with the acyl chloride which might be generated *in situ* from the reaction of the carboxylic acid with POCl_3_. Followed by cyclization and condensation of the intermediate, the target products were formed. The reaction went smoothly by controlling the amount of POCl_3_, and the products were obtained in good yields. The results were presented in Table 1.

**Table 1:** N-substituted prazolo[3, 4-*d*]pyrimidin-4-one **2a-j** via Scheme 3.

Entry	R^2^	R^1^	Yield a	Time(h)
**2 a**	H	2,6-Cl_2_-4-CF_3_-C_6_H_2_-	90	1.5
**2 b**	CH_3_	2,6-Cl_2_-4-CF_3_-C_6_H_2_-	87	2
**2 c**	CH_2_CH_3_	2,6-Cl_2_-4-CF_3_-C_6_H_2_-	90	2.5
**2 d**	CCl_3_	2,6-Cl_2_-4-CF_3_-C_6_H_2_-	89	2
**2 e**	CH_3_	4-OCH_3_-C_6_H_4_-	83	1
**2 f**	CH_3_	2,4-(NO_2_)_2_-C_6_H_3_-	90	1.5
**2 g**	CH_3_	2,4,6-Cl_3_-C_6_H_2_-	97	1.5
**2h**	CH_3_	2-Cl-C_6_H_4_-	82	2
**2 i**	CH_3_	H	75	2.5
**2 j**	CH3	n-Bu	70	2.5
^a^ isolated yields based on compound 2				

**Figure 1:**
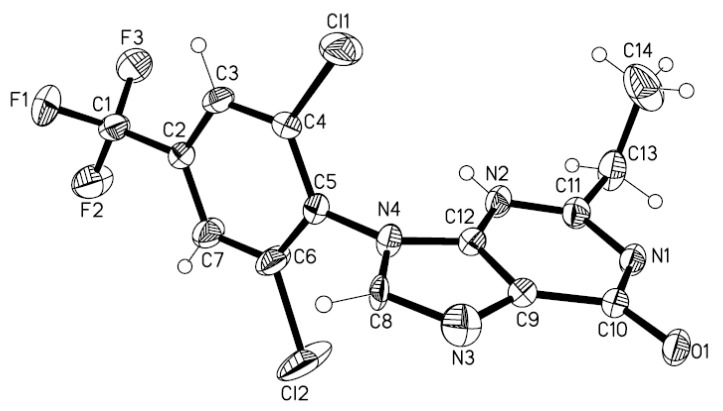
Single crystal X-ray crystal structure of **2c**.

The structures of compounds **2a-j** were deduced from their elemental analyses and their IR, ^1^H- NMR, ^13^C-NMR and mass spectra and all elemental and spectral data of compounds **2a-j** were in accord with the suggested structures. The ^1^H-NMR spectrum of **2c**, as an example, consisted of a singlet at δ 11.06 from the NH function, a singlet at δ 8.27 is from the H-3 proton, a singlet at δ 8.11 due to the phenyl ring (two protons), a multiplet at δ 2.74 (two protons) from the CH_2_ and a triplet at δ 1.23 due to the methyl group (three protons). Moreover the structure of **2c** was confirmed via X-ray crystallographic analysis (Figure 1).

## 3. Experimental

### 3.1. General

All the melting points were uncorrected. ^1^H-, ^13^C-, and ^19^F-NMR spectra were recorded on a FT-Bruker AT-300 instrument using CDCl_3_ or CD_3_COCD_3_ as a solvent with tetramethylsilane (TMS) as the internal standard. J-values are given in Hz. Compounds were properly characterized by elemental analyses using a Carlo-Erba EA-1112 instrument. IR spectra were measured on a Bruker VECTOR55 instrument. Silica gel 60 GF254 was used for analytical and preparative TLC.

### 3.2. General procedure for the preparation of the pyrazolo[3,4-d]pyrimidines ***2a-2j***: preparation of 2c

5-Amino-1-[2,6-dichloro-4-(trifloromethyl)phenyl]-1*H*-pyrazole-4-carbonitrile (0.321 g, 1 mmol) was dissolved in propanoic acid (3 mL). Then POCl_3 _(0.2 mL) was added quickly. The mixture was refluxed for 2 h (the reaction system was carefully observed by TLC). After the mixture was cooled, added ice water (50 mL). A mass of white precipitate was produced. K_2_CO_3_ was added to neutralize the acid till no bubble occurs. The reaction mixture was filtered, and washed with a small amount of ethanol, dried. A 90% yield of the compound was obtained. Crystals of **2c** suitable for X-ray diffraction were obtained by slow evaporation of ethanol-acetone mixture solution. The other compounds were also synthesized according to this method.

*1-(2,6-Dichloro-4-(trifluoromethyphenyl]-4,5-dihydro-1H-pyrazolo[3,4-d]pyrimidin-4-one *(**2a**): White solid; mp 271-273 ºC, IR (KBr, cm^-1^): 3849, 3749, 2924, 1699, 1592, 681;^ 1^H-NMR (CD_3_COCD_3_, 300 MHz): δ 11.32 (s, 1H), 8.36 (s, 1H), 8.15 (s, 1H), 8.12 (s, 2H); ^13^C-NMR (CD_3_COCD_3_, 75 MHz): δ 106.3 (1C), 122.4 (q, *J* = 272 Hz, 1C), 126.4 (1C), 132.7 (q, *J* = 33.75 Hz, 1C), 135.5 (2C), 136.1 (1C), 137.8 (2C), 149.8 (1C), 153.8 (1C), 157.0 (1C); MS: *m/z* (%) = 348 (100, [M^+^]). Anal. Calcd. for C_12_H_5_Cl_2_F_3_N_4_O: C, 41.29; H, 1.44; N, 16.05. Found: C, 41.20; H, 1.45; N, 16.00%.

*6-Methyl-1-(2,6-dichloro-4-trifluoromethylphenyl)-4,5-dihydro-1H-pyrazolo[3,4-d]pyrimidin-4-one* (2b): White solid; mp 259-260 ºC, IR (KBr, cm^-1^): 3772, 3105, 2896, 1598, 1392, 1317, 1131; ^1^H-NMR (DMSO-d_6_, 300 MHz): δ 12.42 (s, 1H), 8.40 (s, 1H), 8.27 (s, 2H), 2.31 (s, 3H);^ 13C^-NMR (DMSO-d_6_, 75 MHz): δ 21.2 (1C), 104.3 (1C), 122.2 (q, *J* = 272 Hz, 1C), 126.4 (1C), 132.6 (q, *J *= 33.70 Hz, 1C), 135.6 (2C), 136.3 (1C), 137.6 (2C), 154.5 (1C), 157.6 (1C), 159.8 (1C); MS: *m/z* (%) = 361 (100, [M^+ ^- 1]); Anal. Calcd for C_13_H_7_Cl_2_F_3_N_4_O: C, 43.00; H, 1.94; N, 15.43. Found: C, 42.91; H, 1.90, N, 15.38.

*6-Ethyl-1-(2,6-dichloro-4-trifluoromethylphenyl)-4,5-dihydro-1H-pyrazolo[3,4-d]pyrimidin-4-one *(2c): White solid; mp 232-233 ºC, IR (KBr, cm^-1^): 3094, 2989, 1681, 1598, 1531, 1319, 1173, 1124, ^1^H-NMR (CD_3_COCD_3_, 300 MHz): δ 11.16 (s, 1H), 8.27 (s, 1H), 8.11 (s, 2H), 2.74 (q, *J* = 7.5 Hz, 2H), 1.23 (t, *J* = 7.5 Hz, 3H);^ 13C^-NMR (DMSO-d_6_, 75 MHz): δ 11.8 (1C), 27.8 (1C), 104.6 (1C), 122.4 (q, *J* = 272 Hz, 1C), 126.5 (1C), 132.7 (q, *J*= 33.75 Hz, 1C), 135.7 (2C), 136.5 (1C), 137.7 (2C), 154.6 (1C), 158.0 (1C), 164.1 (1C); MS: *m/z* (%) = 375 (100, [M^+ ^- 1]); Anal. Calcd for C_14_H_9_Cl_2_F_3_N_4_O: C, 44.59; H, 2.41; N, 14.86. Found: C, 44.51; H, 2.36, N, 14.83.

*6-Trichloromethyl-1-(2,6-dichloro-4-trifluoromethylphenyl)-4,5-dihydro-1H-pyrazolo[3,4-d]- pyrimid- in-4-one *(2d): White solid; mp 238-239 ºC, IR (KBr, cm^-1^): 3013, 2920, 1683, 1589, 1333, 1317, 1124, 663,^ 1^H-NMR (DMSO-d_6_, 300 MHz): *δ* 12.50 (s, 1H), 8.45 (s, 1H), 8.24 (s, 2H);^ 13C^-NMR (DMSO-d_6_, 75 MHz): 79.0 (1C), 105.6 (1C), 122.6 (q, J = 273 Hz, 1C), 126.8 (1C), 132.9 (q, J = 33.75 Hz, 1C), 136.0 (2C), 137.0 (1C), 138.1 (2C), 155.3 (1C), 159.0 (1C), 164.7 (1C); MS: *m/z* (%) = 463 (100, [M^+ ^- 1]); Anal. Calcd for C_13_H_4_Cl_5_F_3_N_4_O: C, 33.47; H, 0.86; N, 12.01. Found: C, 33.451; H, 0.85, N, 12.05.

*6-Methyl-1-(4-methyloxyphenyl)-4,5-dihydro-1H-pyrazolo[3,4-d]pyrimidin-4-one *(2e): White solid, mp 258-260 ºC; IR (KBr, cm^-1^): 3850, 3745, 3618, 2926, 1690 (s), 1518, 1463, 675 ; ^1^H-NMR (DMSO-d_6_, 300 MHz): δ 12.23 (s, 1H), 8.19, (s, 1H), 7.86 (d, *J* = 7.5 Hz, 2H), 7.08 (d, *J* = 7.5 Hz, 2H), 3.80 (s, 3H), 2.37 (s, 3H);^ 13C^-NMR (DMSO-d_6_, 75 MHz): δ 21.5 (1C), 55.5 (1C), 105.2 (1C), 114.3 (2C), 123.5 (2C), 131.5 (1C), 135.3 (1C), 152.1 (1C), 157.9 (1C), 158.1 (1C), 158.3 (1C); MS: *m/z* (%) = 255 (100, [M^+ ^- 1]); Anal. Calcd for C_13_H_12_N_4_O_2_: C, 60.93; H, 4.72; N, 21.86. Found: C, 60.88; H, 4.68, N, 21.76.

*6-Methyl-1-(2,4-dinitrophenyl)-4,5-dihydro-1H-pyrazolo[3,4-d]pyrimidin-4-one* (2f): Yellow solid, mp 229-230ºC; IR (KBr, cm^-1^): 3749, 2921, 1695(s), 1605, 1533, 1348 ; ^1^H-NMR (DMSO-d_6_, 300 MHz): δ 12.52 (s, 1H), 8.85 (s, 1H), 8.70 (d, *J* = 9 Hz, 1H), 8.37 (s, 1H), 8.19 (d, *J* = 9 Hz, 1H), 2.44 (s, 3H); ^13C^-NMR (DMSO-d_6_, 75 MHz): δ 21.5 (1C), 105.6 (1C), 121.3 (1C), 128.5 (1C), 128.8 (1C), 134.1 (1C), 138.4 (1C), 143.3 (1C), 146.1 (1C), 153.9 (1C), 157.5 (1C), 160.1 (1C); MS: *m/z* (%) = 315 (100, [M^+ ^- 1]); Anal. Calcd for C_12_H_8_N_6_O_5_: C, 45.58; H, 2.55; N, 26.58. Found: C, 45.45; H, 2.50, N, 26.46.

*6-Methyl-1-(2,4,6-trichlorophenyl)-4,5-dihydro-1H-pyrazolo[3,4-d]pyrimidin-4-one* (2g): White solid, mp 236-237 ºC; IR (KBr, cm^-1^): 3432, 1685, 1599, 1536, 1386, 667; ^1^H-NMR (DMSO-d_6_, 300 MHz): δ 12.4 (s, 1H), 8.3 (s, 1H), 8.0 (s, 2H), 2.3 (s, 3H);^ 13C^-NMR (DMSO-d_6_, 75 MHz): δ 21.4 (1C), 104.4 (1C), 129.2 (2C), 132.2 (1C), 135.4 (2C), 136.4 (1C), 137.4 (1C), 154.6 (1C), 1587.9 (1C), 159.7 (1C); MS: *m/z* (%) = 327 (100, [M^+ ^- 1]); Anal. Calcd for C_12_H_7_Cl_3_N_4_O: C, 43.73; H, 2.14; N, 17.00. Found: C, 43.67; H, 2.10, N, 16.88.

*6-Methyl-1-(2-chlorophenyl)-4,5-dihydro-1H-pyrazolo[3,4-d]pyrimidin-4-one *(2h): White solid, mp 217-219 ºC; IR (KBr, cm^-1^): 3840, 3745, 2929, 1693, 1602, 1520; ^1^H-NMR (DMSO-d_6_, 300 MHz): δ 12.3 (s, 1H), 8.2 (s, 1H), 7.6 (m, 4H), 2.3 (s, 3H); ^13^C-NMR (DMSO-d_6_, 75 MHz): δ 21.2 (1C), 104.2 (1C), 128.1 (1C), 130.2 (2C), 131.2 (1C), 131.4 (1C), 134.9 (1C), 136.0 (1C), 153.9 (1C), 157.9 (1C), 158.8 (1C); MS: *m/z* (%) = 259 (100, [M^+ ^- 1]); Anal. Calcd for C_12_H_9_ClN_4_O: C, 55.29; H, 3.84; N, 21.49. Found: C, 55.12; H, 3.80, N, 21.36.

*6-Methyl-4,5-dihydro-1H-pyrazolo[3,4-d]pyrimidin-4-one *(2i): White solid, mp 264-265 ºC; IR (KBr, cm^-1^): 3842, 2925, 2272, 1741, 1645, 1518, 1461, 1391, 1121, 669; ^1^H-NMR (DMSO-d*6*, 300 MHz): *δ*12.03 (s, 1H), 10.36 (s,1H), 8.33 (s, 1H), 2.36 (s, 3H); ^13C^-NMR (DMSO-d_6_, 75 MHz): 22.0(1C), 105.00 (1C), 135.17 (1C), 153.70 (1C), 158.78 (1C), 159.20 (1C); MS: *m/z* (%) = 149 (100, [M^+ ^- 1]); Anal. Calcd for C_6_H_6_N_4_O: C, 48.00; H, 4.03; N, 37.32. Found: C, 47.95; H, 4.00, N, 37.28.

*6-Methyl-1-n-butyl-4,5-dihydro-1H-pyrazolo[3,4-d]pyrimidin-4-one *(2j)**,** White solid, mp 144-145 ºC; IR (KBr, cm^-1^):2925, 2855, 1387, 1120, 676; ^1^H-NMR (CD_3_COCD_3_): *δ* 11.60 (s, 1H), 8.44 (s, 1H), 4.20 (t, *J* = 6.8 Hz, 2H), 2.26 (s, 3H), 1.78 (m, *J *= 10.6 Hz, 2H), 1.20 (m, *J* = 7.41Hz, 2H), 0.86 (t, *J* = 7.4 Hz, 3H);^ 13C^-NMR (CD_3_COCD_3_): 13.36 (1C), 19.09 (1C), 21.43 (1C), 31.50 (1C), 52.1 (1C), 104.73 (1C), 128.46 (1C), 155.52 (1C), 159.24 (1C), 159.32 (1C); MS: *m/z* (%) = 205 (100, [M^+ ^- 1]); Anal. Calcd for C_10_H_14_N_4_O: C, 58.24; H, 6.84; N, 27.16. Found: C, 58.20; H, 6.80, N, 27.10.

### 3.3. X-ray crystallography

Compound **2c** was subjected to single crystal X-ray crystallography and intensity data were collected at 298(2)K on an Siemens P4 diffractometer and use graphite Monochromated MoK_a _adiation (λ = 0.71073Å). The structure was solved by a direct method using the SHELXL-97 program [16] and refined with the SHELXL-97 program. All H atoms bonded to the C atoms were placed geometrically at the distances of 0.93–0.96Å and included in the refinementin riding motion approximation with U_iso_ (H) = 1.2 or 1.5U_eq_ of the carrier atom. The thermal ellipsoids were plotted with the SHELXL-97 program at 50% probability. The molecular structure is shown in Figure 1. Selected crystal data and structure refinement details are presented in Table 2. Selected bond distances and angles are listed in Table 3. 

CCDC 774536 contains the supplementary crystallographic data for this paper. These data can be obtained free of charge from the Cambridge Crystallographic Data Centre, 12, Union Road, Cambridge, CB2 1EZ, UK; E-mail: deposit@ccdc.cam.ac.uk.

**Table 2:** Crystal data and structure refinement for C_14_H_9_Cl_2_F_3_N_4_O.

Empirical formula	C_14_ H_9_ Cl_2_ F_3_ N_4_ O
Formula weight	377.15
Temperature	298(2) K
Wavelength	0.71073 A
Crystal system	Monoclinic
space group	P 2/n
Unit cell dimensions	a = 13.468(4) A alpha = 90 deg.
	b = 8.234(3) A beta = 112.056(6) deg.
	c = 15.047(5) A gamma = 90 deg
Volume	1546.4(9)A^3^
Z	4
Absorption coefficient	0.463 mm^-1^
F(000)	760
Theta range for data collection	2.47° to 25.02°
Limiting indices	-16<=h<=15, -9<=k<=9, -17<=l<=14
Reflections collected / unique	7730 / 2740 [R(int) = 0.0213]
Completeness to theta = 25.02	99.6%
Absorption correction	Semi-empirical from equivalents
Max. and min. transmission	0.9214 and 0.8154
Refinement method	Full-matrix least-squares on F^2 ^
Data / restraints / parameters	2740 / 0 / 218
Goodness-of-fit on F^2	1.142
Final R indices [I>2sigma(I)]	R1 = 0.0866, wR2 = 0.2087
R indices (all data)	R1 = 0.0945, wR2 = 0.2142
Largest diff. peak and hole	0.660 and -0.897 e.A^-3 ^

**Table 3:** Selected bond distances (Å) and angles (°) for compound **2c**.

F(1)-C(1)	1.341(6)	O(1)-C(10)	1.236(5)	N(1)-C(11)	1.369(6)
N(2)-C(12)	1.355(6)	N(3)-C(8)	1.310(6)	N(4)-C(8)	1.368(6)
C(1)-C(2)	1.504(7)	C(2)-C(7)	1.360(7)	C(4)-C(5)	1.388(6)
C(6)-C(7)	1.377(7)	C(9)-C(10)	1.436(6)	C(9)-C(12)	1.388(6)
C(11)-N(1)-C(10)	125.1(4)	C(8)-N(3)-C(9)	110.2(4)	C(12)-N(4)-C(5)	127.9(4)
C(8)-N(4)-C(5)	120.2(3)	C(7)-C(2)-C(3)	120.3(4)	C(3)-C(2)-C(1)	119.3(4)
C(4)-C(3)-C(2)	119.8(4)	C(6)-C(5)-C(4)	117.3(4)	C(6)-C(5)-N(4)	120.3(4)
N(3)-C(8)-N(4)	106.8(4)	C(12)-C(9)-C(10)	117.6(4)	N(1)-C(10)-C(9)	112.1(4)
N(2)-C(11)-N(1)	123.9(4)	N(1)-C(11)-C(13)	115.3(4)	N(2)-C(12)-C(9)	127.9(4)

## 4. Conclusions

In summary, we have successfully developed a simple and efficient method for the synthesis of variously functionalized pyrazolo[3,4-d]pyrimidin-4-ones by heteroannulation under mild conditions using POCl_3_. This works has been patented [17]. Further heteroannulation studies are underway in our laboratory.
